# Alternative RNA Splicing—The Trojan Horse of Cancer Cells in Chemotherapy

**DOI:** 10.3390/genes12071085

**Published:** 2021-07-18

**Authors:** Nikolay Mehterov, Maria Kazakova, Yordan Sbirkov, Boyan Vladimirov, Nikolay Belev, Galina Yaneva, Krassimira Todorova, Soren Hayrabedyan, Victoria Sarafian

**Affiliations:** 1 Department of Medical Biology, Medical University-Plovdiv, 4002 Plovdiv, Bulgaria; nikolay.mehterov@mu-plovdiv.bg (N.M.); mariya.kazakova@mu-plovdiv.bg (M.K.); yordan.sbirkov@mu-plovdiv.bg (Y.S.); 2 Research Institute, Medical University-Plovdiv, 4002 Plovdiv, Bulgaria; 3 Department of Maxillofacial Surgery, Medical University-Plovdiv, 4002 Plovdiv, Bulgaria; boyan.vladimirov@mu-plovdiv.bg; 4 Medical Simulation and Training Center, Medical University-Plovdiv, 4002 Plovdiv, Bulgaria; nikolai.belev@mu-plovdiv.bg; 5 Department of Biology, Faculty of Pharmacy, Medical University of Varna, 9002 Varna, Bulgaria; galina.yanevaa@gmail.com; 6 Laboratory of Reproductive OMICs Technologies, Institute of Biology and Immunology of Reproduction, Bulgarian Academy of Sciences, 1113 Sofia, Bulgaria; krasiot@abv.bg (K.T.); shayrabedyan@ibir.bas.bg (S.H.)

**Keywords:** alternative splicing, splice variants, cancer pathobiology, drug resistance

## Abstract

Almost all transcribed human genes undergo alternative RNA splicing, which increases the diversity of the coding and non-coding cellular landscape. The resultant gene products might have distinctly different and, in some cases, even opposite functions. Therefore, the abnormal regulation of alternative splicing plays a crucial role in malignant transformation, development, and progression, a fact supported by the distinct splicing profiles identified in both healthy and tumor cells. Drug resistance, resulting in treatment failure, still remains a major challenge for current cancer therapy. Furthermore, tumor cells often take advantage of aberrant RNA splicing to overcome the toxicity of the administered chemotherapeutic agents. Thus, deciphering the alternative RNA splicing variants in tumor cells would provide opportunities for designing novel therapeutics combating cancer more efficiently. In the present review, we provide a comprehensive outline of the recent findings in alternative splicing in the most common neoplasms, including lung, breast, prostate, head and neck, glioma, colon, and blood malignancies. Molecular mechanisms developed by cancer cells to promote oncogenesis as well as to evade anticancer drug treatment and the subsequent chemotherapy failure are also discussed. Taken together, these findings offer novel opportunities for future studies and the development of targeted therapy for cancer-specific splicing variants.

## 1. Alternative Splicing in Physiological and Neoplastic Processes

The discovery of split genes by Philip Sharp and Richard Roberts not only deserved the Nobel prize for physiology and medicine in 1993 but also played a fundamental role to basic science and medicine [1]. The fact that genes of higher organisms are present in several distinct segments along the DNA molecule opened the door to the discovery of the splicing process. RNA splicing is an important source of protein diversity as the pre-mRNA transcribed from one gene can lead to different mature mRNA molecules that generate multiple functional proteins. In constitutive splicing introns are removed and exons are joined in the order in which they appear in a gene. A breakthrough in modern biology is the finding of alternative splicing (AS), where a deviation from this preferred sequence occurs and certain exons are skipped, resulting in multiple mRNAs isoforms with different functions. Thus, several gene products are generated from a single gene.

Recent knowledge on AS highlights its essential role both in human physiology and pathology. As a co- and post-translational mechanism of gene expression AS allows the generation of more than one mature mRNA transcript from a single gene. It is regarded as a critical evolutionary conserved instrument that contributes to proteome complexity as around 40% out of all ~20,000 human protein coding genes generate multiple protein isoforms [2,3]. Genome-wide studies reveal that 90–95% of human genes encode pre-mRNAs that are alternatively spliced [4]. Additionally, the number of mRNA isoforms encoded by a single gene can vary from two to several [5]. The first findings indicating the presence of differentially spliced genes came from the discovery that the same gene codes for both the secreted and membrane-bound isoforms of immunoglobulins [6].

Afterwards, the same process was shown for a number of mammalian proteins. The splicing reaction includes the excision of noncoding introns and joining together of exons in pre-mRNA to form the mature mRNA transcript. The basic role in this process plays the spliceosome—a supramolecular structure which is a dynamically assembled complex comprising of more than 100 core spliceosomal proteins and five snRNAs that catalyze the stepwise splicing reactions. Numerous auxiliary splicing factors specifically recognize regulatory cis-elements in pre-mRNAs to additionally regulate AS by mainly affecting spliceosomal assembly [7,8].

**Figure 1:**
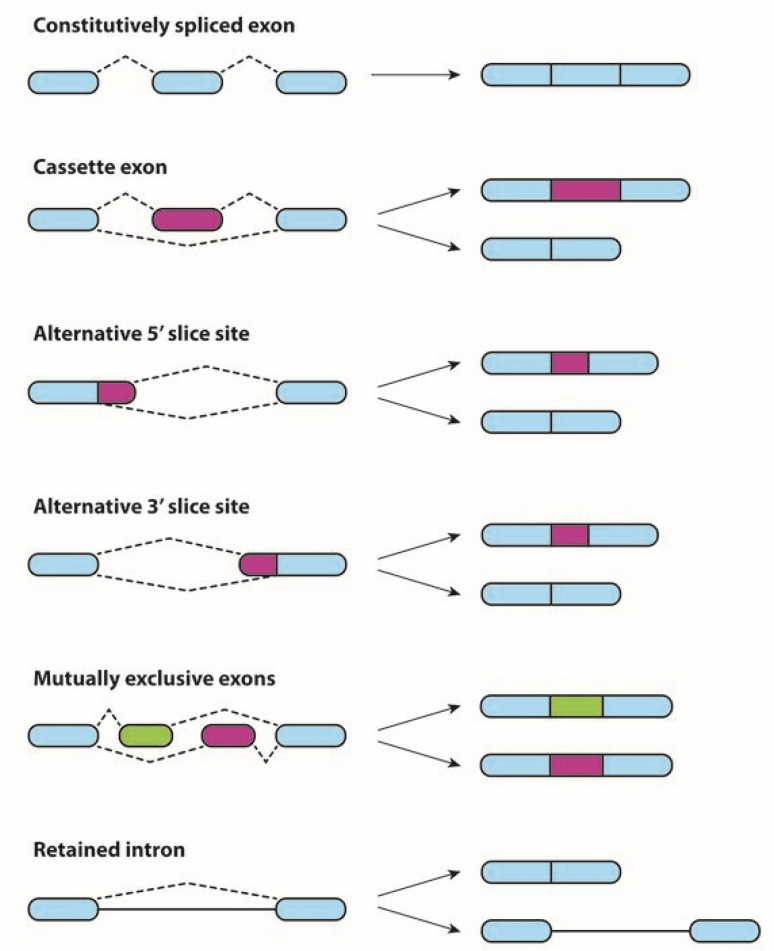
Different types of AS events. Constitutive splicing, exon skipping, alternative 5′ and 3′ sites, mutually exclusive exons, and intron retention are shown. The pre-mRNAs are shown above the arrows; the mature mRNA variants following AS are shown below the arrows.

AS involves the differential use of specific sites to generate protein diversity. The basic mechanisms include exon skipping, choice between mutually exclusive exons, use of alternative splice sites, and intron retention. Exon skipping is a form of RNA splicing used to cause cells to “skip” over defective or misaligned sections of genetic code, leading to a truncated but still functional protein. Mutually exclusive exons are characterized by the splicing of exons in a coordinated manner—one out of two exons (or one group out of two exon groups) is retained, while the other one is spliced out [9]. Additionally, alternative 3′ splice junctions are used, changing the 5′ boundary of the downstream exon. A sequence may be spliced out as an intron or retained. This is distinguished from exon skipping because the retained sequence is not flanked by introns [10]. All these basic molecular mechanisms are related to drug resistance and are presented on Figure 1.

AS is performed in a tempo-spatial-dependent manner and is regulated by numerous cis-acting and trans-acting factors [4]. It is essential to consider that not all alternatively spliced transcripts result in the production of functional proteins. AS promotes a number of physiological events like cell differentiation, lineage determination, tissue and organ development, angiogenesis, etc. [11,12,13]. The transition from embryonic to adult organ structure and functions is based on crucial cell fate decisions most of them resulting from physiological splicing networks. They contribute to the normal development of human brain, skeletal and cardiac muscles, pancreas, liver, as well as to erythropoiesis and germline and immune cell maturation, etc. There is evidence for interchanges between alternatively spliced isoforms during human development which are still not entirely functionally understood [13]. Some alternatively spliced variants occur also in response to external stimuli such as activation of signal transduction cascades [14] and depolarization of neurons [15]. AS might impair RNA stability and localization, as well as further protein synthesis [16].

The misregulation of splicing networks and mutations affecting splicing may well result in different organ pathologies including cancer. The commencement and progression of tumorigenesis are due to gene mutations within the canonical RNA splicing sites or to alterations in the expression level of spliceosomal or splicing regulatory factors [17]. The basic processes which determine the progression and outcome of the neoplastic disease—invasion, metastasis, and angiogenesis, are also subjected to AS variations. They are involved in the initiation, as well as in the evolution of cancer. The first cellular changes related to altered proliferation, differentiation, and apoptosis of tumor cells are regulated by the alternative expression of several oncogenic and tumor suppressor genes [18,19].

Unlike normal cells, in cancer cells a substantial number of aberrant transcripts is accumulated, due to splicing process perturbation and ineffective nonsense-mediated mRNA decay (NMD). NMD is part of the mRNA quality control system. It is responsible for aberrant transcripts degradation, which is enforced by a constant scan of each transcript by the NMD factors for mRNAs harboring premature translation termination codons. In cancer cells, the NMD regulation is also frequently disrupted, conferring aberrant transcripts accumulation [20,21]. Invasion and metastasis are the hallmarks of malignant tumors and depend on the interactions between cancer cells and the tumor microenvironment. The abnormally activated epithelial-mesenchymal transition is influenced by TGF-β which induces AS of the TGF-β-activated kinase 1 [22]. CD44 is another alternatively spliced molecule which initiates the same process in breast cancer cells [23]. A basic molecule implicated in tumor angiogenesis is the vascular endothelial growth factor A (VEGF-A) which might have both angiogenic and anti-angiogenic effects depending on the splicing variants acting on tumor endothelial cells [24]. VEGF-A expression is upregulated via a splice variant of the glioma-associated oncogene homolog 1 thus resulting in boosting angiogenesis in glioblastoma [25].

**Figure 2:**
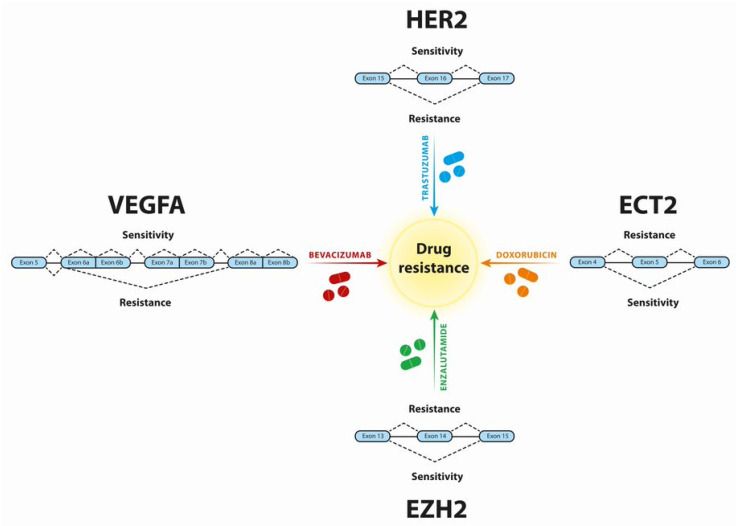
Examples of AS events leading to cancer drug resistance. The AS of HER2, ECT2, EZH2, and VEGFA conferring resistance or sensitivity, respectively, to Trastuzumab, Doxorubicin, Enzalutamide, and Bevacizumab is shown.

Some representative AS events leading to cancer drug resistance are discussed further in the text and summarized in Figure 2.

Another fundamental characteristic of tumor cells is their overgrowth associated with metabolic alterations. The preferential activation of the aerobic glycolysis pathway involves a number of enzyme coding genes prone to AS [26]. Cellular response to hypoxia, especially in breast cancer, is also regulated by AS events [27]. The elucidation of the molecular mechanisms behind AS opens perspectives for new therapeutics to combat human diseases by correcting mis-splicing. Different classes of molecules like splice switching antisense oligonucleotides bind directly to RNA and re-direct AS, while small molecules amend improper kinase activities and spliceosome dysregulation [28]. Drug resistance leading to treatment failure, still remains a major challenge for current cancer therapy. Deciphering the alternative RNA splicing variants in tumor cells would provide opportunities for designing novel therapeutics to combat cancer more efficiently.

The aim of the present review is to offer a comprehensive outline of the recent findings in AS in the most common neoplasms, including lung, breast, prostate, head and neck squamous cell carcinoma (HNSCC), glioma, colon and blood malignancies. The main focus is on the molecular mechanisms acquired by cancer cells to promote oncogenesis and to evade anticancer drug treatment. The perspectives for the development of novel targeted therapies for specific cancer splicing variants are discussed.

## 2. Lung Cancer

Lung cancer remains the leading cause of cancer morbidity and mortality, with more than 2.1 million new cases and 1.8 million deaths reported recently worldwide [29]. Approximately 85% of patients can be classified as the histological subtype known as non-small-cell lung cancer (NSCLC). The most prevalent subtypes are lung adenocarcinoma (LUAD) and lung squamous cell carcinoma (LUSC) [30,31].

There are different mechanisms of eliciting aberrant transcripts and increased tumorigenesis in lung cancer. The most frequent ones are associated with mutations in nonsense-mediated mRNA decay factors (NMD factors). The most commonly mutated NMD factor—UPF1—when functionally inhibited, leads to deposition of aberrant transcripts in the tumor. Another mechanism involves impaired gene expression of splicing-related factors. Commonly, the core components of spliceosomes, U2AF1 and SF3B1, are altered in cancer. It was proved that some specific irregular isoforms clearly promote oncogenesis. Recent combined long and short sequencing of 22 lung cancer cell lines and patients’ tissue specimens showed several aberrant transcript isoforms. They include unannotated exon, exon skipping, exon shuffling, intron retention, alternative first exon, alternative last exon, alternative 5′ splice site, and alternative 3′ splice site [20]. Additionally, novel combinations of several splicing events are also frequently found on a single full-length transcript. Nanopore long read sequencing have provided an immense advantage in finding these new transcripts that were previously impossible to discern based on short read sequencing. These new patterns account for 13.6% of the unannotated cancer *de novo* isoforms, with characteristic NSCLC aberrant splicing events being fairly distinct among cancer specimens. Many genes produce multiple isoforms, each having different AS [20]. On the other hand, repetitive element loci in splicing sites, which can potentially lead to misalignment of the reads, were not found linked to novel splice sites [20]. 5. Gene-fusion events were also reported to function as driver genes contributing to tumorigenicity in many cancers [32,33], such as newly identified in lung cancer fusion transcripts EML4-ALK, and ERGIC2-CHRNA6. The latter is a drastic frameshift fusion event causing an amino acid change that had actually four alternative splice isoforms, consisting of an unannotated exon and an alternative last exon [20]. NMD is a key mechanism for elimination of aberrant splicing isoforms. UPF1 of the NMD system is found often harboring splice site mutations itself. Its experimental or cancer induced silencing results in an immediate NMD clearance inefficiency. It is demonstrated by spliceosome SURF2 gene intron retention and an increased exon 2 alternative 5′ splice site [20], ignoring premature translation termination codon presence. Besides NMD factors, spliceosome key factors such as SF3B1 are also affected. This is a key splicing factor that is mutated in several diseases and causes the increase of aberrant splicing isoforms. SF3B1 perturbation or gene silencing results in significant increase in the exon skipping and intron retention. When one of the most common hotspot mutations located in the HEAT-repeat domain of SF3B1-K700E occurred, the intron retention was preferentially replaced with alternative 3′ splice site events [34].

Genes with exon-skipping isoforms show significant enrichment in the translation and ubiquitin-proteasome pathways. PSMD7, a 19S proteasome subunit, overexpressed in most carcinoma cells [35], was recently associated with resistance to conventional chemotherapy, and exon 3 and 6 skipping. It most likely plays a critical role in cancer immune escape [20]. Using long read sequencing, most of the lung cancer specimens were found to harbor most frequently either frameshifts, or alternative last exon or 5′ splice sites, all resulting in the expression of new putative antigens. Frameshifts are harbored in cancer retained NMD-targeted isoforms, but also in key NMD factors, as UPF3B and SMG8. These findings suggest that the NMD pathway perturbation is a key mechanism for AS aberrations genesis and enrichment [20]. This potential immunogenic neo-antigen peptide sources in cancer are opposed by a powerful mechanism of “coordinated” rise of immune evasion promoting aberrant isoforms. Aberrant transcript changes specific for the most common histological NSCLC subtypes—LUAD and LUSC—were discovered. Several cancerogenesis promoting perturbed pathways, harboring aberrant transcripts genes, were detected. The integrin A9B1 pathway was found to be significantly enriched in males with LUAD. This pathway has an important regulatory effect on the induction of pro-survival and pro-proliferative signaling cascades, while in female LUAD, the MYC repression was enriched. It is playing an important role in cellular proliferation, differentiation, apoptosis, and cell cycle progression. On the other hand, in male LUSC, VEGFR1/2 and FAK pathways were enriched, while in female LUSC, the p53 binding pathway was enriched. It was shown that 3691 and 2403 AS events were significantly associated with patient survival and drug resistance in LUAD and LUSC, respectively [36].

The RNA binding motif protein 10 (RBM10) is a novel putative drug target. It is an auxiliary splicing factor that promotes exon skipping by binding to the adjacent intronic regions [37]. RBM10 was very recently found to be the second most frequently mutated gene, compared to commonly mutated tumor suppressor genes in LUAD patients. RBM10 mutations co-occurred with major oncogenes EGFR and KRAS mutations in 93% and 78% of cases, respectively [37]. These mutations were suggested to have a role in driving LUAD development and progression, and are prevalent in female and younger non-smoking patients, highlighting RBM10 clinical significance. RBM10 is a poor therapeutic target, as its mutations are most often loss-of-function ones. Hence, elucidation of new RBM10-regulated AS targets provides a valuable therapeutic option, like the loss of AS regulation of its key target eukaryotic translation initiation factor 4H (EIF4H). LUAD patients with low RBM10 expression level have reduced survival rates. RBM10 mutation-associated AS events are predictive for LUAD patient survival, with EIF4H exon 5, ELF2 exon 3, MON2 exon 29, and EXOC1 exon 11, all being negatively correlated with survival rates. As a major consequence of RBM10-regulated AS of its key target EIF4H, two alternative splice variants of exon 5 are produced—EIF4H-L and EIF4H-S. EIF4H is a translation initiation regulator, and it is closely linked with cancer. The inclusion of exon 5 in its spliced isoforms renders a highly malignant phenotype. When RBM10 loss of function results in an increased exon 5 harboring EIF4H cancer-specific isoform, the alternatively spliced genes by EIF4H-L are considered as good putative targets [37].

### Anticancer Drug Resistance Based on AS in Lung Cancer

A plethora of different modifications were found to be related to resistance to conventional chemotherapeutics, as well as to targeted agents. These include transformations in factors mediating drug uptake and efflux, conversion of prodrugs to their active metabolites, drug inactivation. A number of qualitative and quantitative alterations in drug targets and drug sequestration within organelles were detected. Apoptosis was also affected. AS and splicing-based defects were shown to contribute to chemotherapy resistance of lung cells to glucocorticoids (GC). Hitherto, numerous splice variants of the GC receptors were reported. Some of them antagonized the function of the canonical isoform GRα in a dominant negative mode [38]. The spliceosome member U2AF1 and multiple HNRNPs were found to be differentially spliced between GC-sensitive and GC-resistant cells [39]. Gemcitabine treatment of cancer cells eventually elevated splicing factor SRSF1, thus inducing an AS of the MAP kinase-interacting serine/threonine-protein kinase 2 (MNK2) gene towards its MNK2b variant. In the exon 13b isoform lacks MAPK binding site, and is uncoupled from upstream MAPK regulation [40]. MNK2b splice variant phosphorylates eIF4E stronger, reducing Gemcitabine-induced apoptosis, promoting cancer cell survival, cell proliferation and ultimately Gemcitabine resistance [41,42]. Genotoxic pro-survival MNK2b/ eIF4E pathway is entirely SRSF1-dependent and SRSF1 induced. A splicing shift in the pyruvate kinase (PKM) gene in favor of PKM2 variant [43] is also able to cause Gemcitabine and Cisplatin resistance in cancer. SRSF1, along with SF3B1, are also involved in apoptosis modulation in cancer through AS. They produce chemotherapy hampering longer anti-apoptotic isoforms by exon 2 inclusion in MCL1 and alternative 5′splicing site recognition in exon 2 of BCL-X. While blockade of SRFS1 with small molecules (i.e., E7107) could restore some of the BCL2 family members to shorter pro-apoptotic variants—BCL-XL was found resistant to this treatment [44]. SRSF1 was also involved in epithelial to mesenchymal transition (EMT) and an increased tumor aggressiveness via its exosome secretion [45].

The oncogenic KRAS correlates with an ETS transcription factor-mediated aberrant-splicing regulatory network. It induces the AS factor polypyrimidine-tract binding protein (PTBP1), resulting in a shift in AS of small GTPase RAC1, the Notch-signaling related adaptor protein NUMB and pyruvate kinase gene isoform 2 (PKM) transcripts in LUSC. These changes are related to a more malignant phenotype with activated Notch, and PKM2 mediated drug-induced genotoxic stress-resistance. Similar to other AS modulators PTBP1 is crucial for PKM2 splicing and short survival, and it is also Gemcitabine induced. Several therapeutic strategies have emerged from the AS phenomenon. The first is related to spliceosome modulation monotherapy using small molecules, derived from *Pseudomonas* sp. or *Streptomyces* sp., that bind directly to SF3B1 protein in the U2 snRNP. Synthetic 2nd generation small molecules are also developed—Spliceostatin A (SSA), Meayamycin B (MAMB), Sudemycins, E7107, and H3B-8800. Mutations in the key side adenosine residues used by spliceosome modulators to bind SF3B1 and PHF5A result in resistance. This is related to the four-protein complex formed by SF3B1, SF3B3, PHF5A, and SF3B5, and the close proximity between PHF5A and E7107 binding site on SF3B1 [46]. The SF3B1 modulators have promising cytotoxic activity, which is enhanced in cancer cells as compared to non-malignant cells, while retaining activity against (multi-) drug resistant cells. The cytotoxic activity is due to a direct effect on the splicing of genes involved in cell cycle and apoptosis regulation, DBA double strand break. The SF3B1 modulator MAMB was shown effective in NSCLC with combination with BCL-XL inhibitor ABT-737 (Navitoclax), inducing splicing shift of MCL1 towards pro-apoptotic MCL1S. The new therapeutic approaches allow tumor types stratification and adaptive inclusion of different inhibitors based on AS mode of action, hence precise medicine intervention.

The affected genes, the mutation type and its relation to drug resistance in lung cancer are presented on Table 1.

## 3. Breast Cancer

The updated estimates on the global cancer burden using the GLOBOCAN 2020 of cancer incidence and mortality produced by the International Agency for Research on Cancer show that female breast cancer is the most frequent malignant neoplasm in women [50]. It surpasses lung cancer as the most commonly diagnosed cancer worldwide. In 2020, there are a total of 2,261,419 new breast cancer cases (or 1.7%) and 684,996 new breast cancer deaths (or 6.9% of all cancer cases).

AS plays a significant role in breast cancer prognosis, survival, and drug resistance; hence, it offers a valuable option as a therapeutic target [51]. Its regulation contributes to anticancer drug resistance by altering the coding region of drug targets [52]. The disruption of canonical splicing governs breast cancer clinical progression [53]. AS generates mainly the glucocorticoid receptor-α isoform important for the regulation of the receptor for activated C kinase 1, a scaffolding protein with a glucocorticoid response element site on its promoter [54]. The transcriptional regulation is achieved by a mechanism connected to the SRSF3 splicing factor. It in turn promotes the glucocorticoid receptor-α, the transcriptional regulation of the receptor for activated C kinase 1, and consequently cell migration. This mechanism is positively regulated by cortisol. The quantitative isoform expression is a potential prognostic and predictive biomarker in estrogen receptor-positive breast cancer [53]. Resistance to endocrine therapy in breast cancer is due to mutations in SF3B1 and upregulation of SF3B3 and SRSF1—basic spliceosomal component genes. Exploring the consequent alternatively spliced transcripts elucidates the mechanism of these modifications and their function in therapy resistance. The expression of the d16HER2 splice variant in human EGFR 2-positive breast cancer has a crucial pathobiological function [55]. This variant significantly influences the susceptibility of breast cancer cells to Trastuzumab when compared with its wild-type counterpart, thus constituting a new and potentially clinically useful biomarker. The aggressiveness, stemness, and Trastuzumab susceptibility of HER2-positive breast cancer is associated with deletion of exon 16 in HER2 splice variant known as d16HER2 [56]. The existence of outliers with d16HER2 expression in breast cancer is associated with a clinical benefit/response to the single agent Trastuzumab. Patients with long-term benefits from Trastuzumab are characterized by high d16HER2 mRNA scores. Among HER2 splice variants in breast cancer, the P100 variant potentially reduces the efficacy of the anti-HER2 therapy with Trastuzumab [57].

The function of SRSF3 in Paclitaxel treatment in breast cancer is analyzed by gain-of-function or loss-of-function assay in the breast cancer cell line MCF-7 [58]. This treatment decreases SRSF3 expression. SRSF3 overexpression rescues the growth inhibition caused by Paclitaxel in breast cancer cells. Increased SRSF3 exon 4 exclusion correlates with poor survival in breast cancer patients. SR-3 downregulates the SRSF3 protein expression and significantly increases cancer cell sensitivity to Paclitaxel treatment. RNA-sequencing enables profiling of alternatively spliced transcripts in breast and in many other cancers [59].

A meta-analytical framework combining the pharmacological data from two large-scale drug screening datasets is developed to identify robust transcriptomic biomarkers for drug response across studies. The analysis of two independent breast cancer datasets reveals that specific isoforms of IGF2BP2, NECTIN4, ITGB6, and KLHDC9 are significantly associated with AZD6244, Lapatinib, Erlotinib, and Paclitaxel, respectively, in multiple screening using different pharmacological assays. Isoform expressions are a rich resource for biomarkers predictive of drug response. A total of 1723 AS variants and 41 splicing factors regulated in a breast cancer cell model of acquired resistance to Doxorubicin are identified [60]. A RNAi screen on splicing factors reveals both ZRANB2 and SYF2, whose depletion partially reverses Doxorubicin resistance. They are associated with ECT2 pre-messenger RNA and further ECT2-exon 5+ isoform depletion reduces Doxorubicin resistance. Moreover, high ECT2-exon 5 inclusion levels are associated with poor prognosis in breast cancer after chemotherapy. The functional consequences of aberrant RNA splicing and several aberrant splice variants in promoting resistance to cancer targeted therapy or immunotherapy are highlighted [61]. The K700E mutation is detected in 1.8% of breast cancers which exhibit alterations in RNA splicing patterns [62]. The IR-A isoform plays a predominant mitogenic role in breast cancer within a novel interplay between the RNA-binding protein CUGBP1 and the insulin receptor gene. It undergoes AS implicating the CUGBP1 and IR-A isoforms as the potential therapeutic targets and biomarkers for breast cancer [63].

The regulatory network of splicing factors and AS events is constructed and DDX39B is identified as the node splicing factor gene [64]. In triple negative breast cancer patients, the exon-specific expression of EPHX2 (exo7), C6orf141 and HERC4 (exo23) shows the presence of specific AS splicing events in these genes. They are associated with different responses to neoadjuvant chemotherapy in comparison to the expression of the parental genes. Further on, the expression of AS events changes dynamically in the progress of chemotherapy treatment and differs among the individuals, underlining that AS is a potential sensitive predictor and target of the treatment [64]. Knockdown of the epithelial splicing regulatory protein 1 in endocrine-resistant breast cancer models significantly decreases growth. It also alters the epithelial-to-mesenchymal transition splicing signature confirmed in estrogen receptor-positive breast cancer [65]. In Tamoxifen-resistant cells, this knockdown affects lipid metabolism and oxidoreductase processes, resulting in decreased expression of fatty acid synthase, stearoyl-CoA desaturase 1, and phosphoglycerate dehydrogenase at both the mRNA and protein levels. The epithelial splicing regulatory protein 1 could form the basis for the prevention of Tamoxifen resistance in this breast cancer [65]. Different expression patterns of lectin-like oxidized low-density lipoprotein receptor and its splice variant Δ4 correlate with breast cancer phenotypes [66]. The overexpression of the splice variant Δ4 of this receptor strongly modulates histone H4 acetylation and Ku70, the limiting factor of DNA double-strand breaks repair machinery which are implicated in apoptosis inhibition and drug resistance acquisition. The complete understanding of this receptor and its splice variant Δ4 in breast cancer molecular phenotypes represents a new challenge for targeting tumor metabolic pathways involved in proliferation and drug resistance acquisition [66]. In immune-competent breast cancer models, spliceosome-targeted therapies cause tumor cell-intrinsic antiviral signaling, downstream adaptive immune signaling, and tumor cell death [67]. Additionally, RNA mis-splicing in human breast cancer correlates with innate and adaptive immune signatures. Nine splicing factors, including SNRPD2, SNRPD3, and NHP2L1, control breast cancer cell proliferation in two triple negative breast cancer cell lines [68]. This mechanism involves effective sororin splicing and thereby appropriate sister chromatid cohesion. Moreover, SUN2 is identified as an important new spliceosome complex interacting with a protein that is critical in this process. The overexpression of the myeloid cell leukemia-1 gene is associated with poor prognosis and drug resistance [68]. In breast cancer cells, its AS results in the expression of two functionally distinct proteins, the anti-apoptotic myeloid cell leukemia-1L (exon 2 included) and the pro-apoptotic myeloid cell leukemia-1S (exon 2 skipped). Transfecting siRNAs that target hnRNP K and the hnRNP F/H family causes a switch in splicing towards the pro-apoptotic myeloid cell leukemia-1S. The targeting of the splicing process of myeloid cell leukemia-1 along with other apoptotic regulators is a new therapeutic target in breast cancer cells and provides an effective way to overcome therapy resistance in these cells [69]. Tip60-mediated acetylation of serine-arginine protein kinase 1 is closely associated with chemotherapy sensitivity [70]. In breast cancer cells, Cisplatin induces this acetylation pattern while in the corresponding resistant cells, it reduces acetylation. Additionally, Cisplatin increases phosphorylation and kinase activity of serine-arginine protein kinase 1 SRPK1, favoring the splicing of some anti-apoptotic variants.

On Table 2 the effects of AS on drug resistance in breast cancer are summarized.

## 4. Prostate Cancer

Prostate carcinoma (PCa) is second to lung carcinoma as the leading cause of oncogenic death in men. Locally advanced and metastatic PCa is generally treated with androgen deprivation therapy (ADT) based on testicular androgen synthesis blockade using luteinizing hormone releasing hormone agonists/antagonists. Tumors that have failed this first-line therapy are termed castration-resistant PCa (CRPC) and are further treated with anti-androgens, such as Enzalutamide (Enza). It interferes with androgen receptor (AR) functions, keeping tumor progression in pace only for few months before recurrence. Although the majority of CRPC are classified as adenocarcinoma (Ad) (CRPC-Ad), up to a quarter of them evolve to an aggressive, AR− indifferent disease with neuroendocrine features (CRPC-NE) [71]. In general, all CRPCs consist of undifferentiated basal, stem-like cells [72,73]. Their lineage plasticity serves as a major driver for enhanced treatment resistance and tumor progression. To date most metastatic CRPC (mCRPC), still remain lethal [74].

Dysregulation in pre-mRNA AS is emerging as a hallmark of cancer [75]. PCa cancer development and progression is associated with isoform switch of genes such as AR, CD44, MEAF6, etc. [76,77]. As nearly all multi-exon human genes undergo AS, this tightly regulated process dramatically expands transcriptome and proteome encoded diversity. Primary PCa (pri-PCa) possesses almost twice more AS events compared to normal tissues. PCa either post-androgen deprivation therapy or post-neoadjuvant hormone therapy, displays an even increased number of differentially spliced events (DSEs). This suggests that the global AS pattern which probably contributes to therapy resistance is subjected to a treatment-induced reshaping. A major driver of AS landscape global shift is most likely the loss of RB1/TP53 tumor suppressors, as a large number of DSEs become present following a lineage switch from AR+ luminal to AR- basal-like cells after RB1/TP53 knockdown [78]. The analysis of DSE showed that Intron retention (IR) was enriched in primary PCa vs normal tissue [74], post-androgen deprivation therapy [79], in CRPC vs pri-PCa (TCGA, SU2C, [74]) and in CRPC-NE vs CRPC-Ad [71]. In terms of following lineage plasticity and cancer progression, IR was also enriched in basal (stem like phenotype) vs luminal (differentiated) phenotype, while exon skipping (SE) and mutually exclusive exons (MX) were decreased among DSE. When lineage plasticity was interrogated experimentally, silencing p53 tumor suppressor [78], then IR was again increased, along with alternative 3′ splice sites (A3) and also alternative 5′ splice sites (A5) to a lesser extent. These findings suggest that DSE and IR act specifically as driver mechanisms in acquiring castration resistant phenotype. In CRPC compared to pri-PCa IR were enriched 800 folds, along with SE, A3 and A5 (TCGA, SU2C) [80]. Similarly, CRPC progression to CRPC-NE phenotype is accompanied by extensive IR and A3, A5 events, all leading ultimately to a therapy resistant phenotype [81]. These data indicate that an elevated IR is a hallmark of PCa aggressiveness and stemness, and that PCa development is accompanied by increased AS events. Furthermore, castration resistance and, in particular, metastases are characterized by supplementary significant increases in AS events [74]. While in normal stemness there is some IR, associated with weak splice sites, in PCa, IR is likely trans-regulated, causing functional expression of these IR-bearing genes [74,78]. In PCa, AR is known to be obligatory for pri-PCa growth and continues to be expressed and functionally important in CRPC [82]. In PCa, AR regulates a splicing program distinct from transcription. ADT promotes a stem-like phenotype in PCa [72] and relapsed tumors often exhibit enhanced stem cell properties [83]. The advance of PCa towards CRPC phenotype is therapeutically controlled using an antiandrogen strategy to block the activity of the AR. Popular antiandrogens like Enzalutamide, antagonizing the interaction of androgens with AR, and Abiraterone, inhibiting androgen biosynthesis, are used [84,85]. As we already discussed, castration resistance most likely arises due to the selective pressure exerted when trying to prevent AR signaling. A splice variant AR-V7 arises by inclusion of a cryptic exon CE3, which deprives AR of its ligand binding domain, eliminating effectively the binding of antiandrogens. Even more, this AR-V7 variant is constitutively active and is associated with poor outcome of the antiandrogen treatment [86,87], as all men acquired resistance to these agents over a period of 12 years. The splicing of the AR-V7 variant is shown to be promoted by the AS regulators nuclear ribonucleoprotein L (HNRNPL) [88], and two other family members—HNRNPA1 and HNRNPH [89]. They all bind and co-regulate AR and induce its variant AR-V7, and also activate Myc, and others, suggesting strong involvement in metastatic PCa progression and drug resistance. Silencing of HNRNPH1 sensitized prostate cancer cells to Bicalutamide and inhibited prostate tumor growth in vivo [88]. Aberrant splicing forms were found to arise either due to splicing factor or splicing site mutation. Sometimes other mechanisms such as overexpression are also involved, as in the case of the newly implicated splicing factor, SF3B2 [75]. High SF3B2 expression, without any mutation, was shown to increase AR-V7 through direct binding to AR cryptic exon 3, conferring an aggressive phenotype [90]. SF3B2 is a component of the SF3B1 complex, known as SF3b [91]. SF3B1/SF3b functions as a component of U2-small nuclear ribonucleoprotein (snRNP) in pre-mRNA splicing [92,93]. It also interacts with U2AF2 to participate in 3′ splice sites [94,95,96], while the interaction with SF3B2 allows control of the inclusion or exclusion of SF3B2-bound exons or introns, respectively. Thus, SF3B2 and U2AF2 have different global binding profiles. SF3B2 depletion affects approximately ¼ of the detected splicing events and increases intron retention (IR) and exon skipping (ES) [97]. Based on that, the use of Pladienolide B, an inhibitor of a splicing modulator of the SF3b complex [98] able to suppress tumor growth, was recently suggested [90]. This potential was demonstrated in two clinical trials of the first-in-class spliceosome inhibitor E7107, a pladienolide derivative, showing controlled tumor growth in approximately 30% of the patients [99,100].

Towards mCRPC progression, genomic amplifications in oncogenes, together with alterations in splicing regulatory genes are found to impact AS events the most, cumulatively increasing PCa aggressiveness [74]. The genome amplified oncogene Myc was implicated as a regulator of AS-coupled nonsense-mediated decay [101]. The incorporation of an alternative poison exon, containing premature translation termination codon, in splicing factor and proto-oncogene SRSF3 [102,103] makes it particularly responsive to Myc [101]. During progression towards mCRPC, Myc signaling leads to an increased exon skipping, reduced NMD, and increased expression of SRSF3 [104,105], similar to SF3B2. The most malignant NEPC phenotype is related to the involvement of cell lineage transdifferentiation [106] mediated by transcriptional repressor of neuronal genes RE-1 silencing transcription factor (REST) [107] or by epigenetic modulators. They include Enhancer of Zeste, polycomb repression complex 2 (EZH2) [108] or Serine/arginine repetitive matrix 4 (SRRM4) [109], and cause dramatic neuronal morphological changes. They accelerate cell proliferation in AR-positive or AR-negative prostate cancer cells, respectively. This results in activating pluripotency gene networks associated with stem-cell like differentiation [110], and the complex network between Myc and EZH2, and REST/SRRM4. It is shown that SRY-Box 2 (SOX2) induces the NEPC phenotype. It should be emphasized that it is now considered that a splicing event is an essential part of this mechanism. SRRM4 promotes NEPC by REST-independent manner, targeting SOX2, promoting lineage plasticity and anti-AR therapy resistance in TP53- and RE-1- deficient prostate cancers [78,111]. EZH2, the catalytic subunit of the polycomb repressor complex 2 (PRC2), promotes cancer development and progression through epigenetic silencing of tumor suppressors by trimethylation on histone H3 (H3K27me3) [112,113]. EZH2 also activates AR transcription by direct promoter binding [114]. Later, the TMPRSS2:ERG fusion causes crucial for cancer progression events, disrupting lineage-specific differentiation of the prostate and potentiating EZH2-mediated dedifferentiation program [113]. ERG disrupts AR signaling by inhibiting both AR expression and AR-targets transcription. It turns on EZH2 repressive epigenetic programs, ultimately resulting in subverting cancer cells to a stem-cell-like epigenetic state [113]. Enzalutamide-resistance in CRPC cells is shown to be associated with EZH2 [115]. The combination of GSK126 and Enzalutamide inhibits proliferation and colony formation of Enzalutamide-resistant CRPC cells in a synergistic manner, where efficacy is further enhanced after EZH2 depletion [116]. GSK126 has opposite effect of EZH2, by inhibiting TMPRSS2 in an AR-independent manner in Enzalutamide-resistant CRPC cells. Markers for epithelial-to-mesenchymal transition like E-cadherin were found decreased in Enzalutamide-resistant cells compared with those in Enzalutamide-sensitive cells [115]. We have previously found miR-204 to regulate TMPRSS2:ERG in fusion harboring cells [117], and to induce AS events such as E-cadherin isotype switching, suggesting for even more complex role of AS and epigenomic regulation in acquiring castration-resistant phenotype [118]. We have also observed multiple genes IR phenomena after miR-204 expression manipulation and in cancer stem cell like enriched phenotype (data not published). AS events and their association with drug resistance in PCa are presented on Table 3.

## 5. Head and Neck Squamous Cell Carcinoma

Head and neck squamous cell carcinoma (HNSCC) remain a malignancy with significant mortality, accounting for up to 600,000 new cases and 380,000 deaths annually [120]. Despite substantial insights into the tumorigenesis of HNSCC and advances in diagnosis and therapy, its 5-year survival rate has not changed in the last decades [121]. It is well known that the high mortality rate associated with HNSCC is largely due to late primary diagnosis. The lack of reliable early biomarkers contributes to this problem as HNSCC is typically detected by visual and tactile examination when the disease becomes symptomatic, often at locally advanced stages [122,123]. Another reason for the significant mortality rates is the chemo- and/or radio-resistance that these tumors originally have or develop later [119,124,125,126,127].

Global genome profiling, represented by coding and non-coding RNA sequencing, revealed that many AS events accompany the initiation and progression of this type of malignancy [128]. Recently, it was demonstrated that most genes undergoing AS in HNSCC participate in apoptosis, DNA repair, mRNA splicing, development, and metabolism [129]. For example, the homeobox TGIF1 gene participates in embryonic development and other members from the same family have altered expression in many solid cancers [130]. The TGIF1 gene has 12 splice isoforms and among them TGIF1v2 and v8 are overexpressed in oral squamous cell carcinoma (OSCC) tissues, whereas TGIF1v5 has low expression. It was shown that the overexpression of TGIF1v8 correlated with lower cellular differentiation, positive vascular blood invasion, advanced pathologic stage, and positive vascular lymphatic invasion of OSCC. All these data highlight the role of TGIF1v8 as an oncogenic splicing variant during oral carcinogenesis [131]. The serine/arginine-rich family proteins are actively involved in pre-mRNA AS site selection [132]. SRSF3 is a proto-oncogene with an abnormal expression in many malignancies, including OSCC [133,134,135,136,137]. Its importance as a gene expression regulator comes from the fact that SRSF3 participates in various biological processes including AS [138], alternative RNA polyadenylation [138], termination of transcription [139], RNA export [140], transcriptome integrity [141], microRNA biogenesis [142], and protein translation [143]. The precise analysis of SRSF3 mRNA showed AS of exon 4. The long isoform with exon 4 encodes a truncated SRSF3 protein, whereas the short isoform lacks exon 4, encoding a full-length SRSF3 protein [144]. The in vitro experiments performed on both OSCC (CAL 27 and SCC-9) and breast cancer (MCF-7) cells revealed that Paclitaxel treatment decreased SRSF3 expression. In contrast, SRSF3 overexpression rescued the growth inhibition caused by the drug. Yang et al. demonstrated that another member of the SRSF family, SRSF5, is subjected to autoregulation controlled by SRSF3 [145]. Furthermore, SRSF3 has the potential to induce oral carcinogenesis through SRSF5.

AS events correlate with the survival of HNSCC patients suggesting that their individual expression as a signature holds prognostic properties. The most prevalent AS events related to survival of HNSCC patients are exon skipping, as well as the use of alternate promoter sites, alternate terminator sites, alternate acceptor sites, and alternate donor sites [128]. Among the differentially expressed AS genes, C5orf30, eEF1A lysine and N-terminal methyltransferase (METTL13), Ras homolog gene family member T1 (RHOT1), ATP-binding cassette sub-family C member 5 (ABCC5), and Myelin protein zero-like protein 1 (MPZL1) were shown to possess clinical significance related to patient survival [146]. In another study an AS-based model with a moderate predictive ability in two study models (AUC = 0.83 and 0.82 for 3- and 5-year OS in the development model; AUC = 0.83 and 0.82 for 3- and 5-year in the bootstrap validation model and 5-fold cross-validation model) was developed [147]. The low-risk patients tended to be more sensitive to MK.2206, EHT.1864 and Nutlin.3a chemo drugs as it was proven by the lower IC50 values. Moreover, the authors discovered a link between five survival-related splicing factors (CELF2, TIA1, HNRNPC, HNRNPK, and SRSF9) and 62 survival-related AS events, suggesting potential prognostic properties.

Adenoid cystic carcinoma (ACC) is the second most common malignancy that originates from the major and minor salivary glands [148]. It is characterized by high metastatic potential and poor response to currently available therapeutic drugs [148]. After RNA sequencing of primary ACC tumors and matched normal salivary gland tissues, three novel, truncated variants of FGFR1 were identified [149]. The same shorter versions (FGFR1v) of FGFR1 were further confirmed in ACC cells and patient-derived xenografts. The three FGFR1v are cell surface proteins involved in the AXL/AKT signaling axis. Their expression makes ACC cells resistant to FGFR1 inhibitors. These results were further supported by the fact that downregulation of endogenous FGFR1v increased the cytotoxic effect of Bemcentinib which is an orally administered AXL inhibitor. The drug resistance associated with AS in HNSCC and OSCC is summarized on Table 4.

## 6. Glioma

Glioma, the intracranial malignant tumors, are classified into four grades according to the World Health Organization (WHO). They are subdivided into diffuse low-grade glioma including grades II and III, and grade IV—glioblastoma multiforme (GBM). GBM is the most common malignant, highly heterogeneous primary brain tumor in adults [150]. It accounted for about 18,020 deaths in the USA from primary brain cancer in 2020 (National Cancer Institute). Recent studies reported that AS may affect oncogenes and apoptosis and could stimulate tumor proliferation [151]. In addition, splicing events play a role in drug resistance and tumor metastasis [52,152]. In the brain, AS provides finely tuned expression of protein isoforms in different brain regions and contributes to both gliomagenesis and aggressiveness. Several studies found that aberrant splicing patterns are involved in glioma tumorigenesis and progression [153,154,155]. A few investigations showed that the initiation of glial brain tumors is influenced by aberrant splice isoforms, including epidermal growth factor receptor (EGFR), tumor protein p53 (TP53), Ki-67 antigen, hnRNPA2/B1, phosphatase, and tensin homolog (PTEN) [156]. The human calcitonin receptor gene (CALCR) encodes a high affinity receptor for the peptide hormone calcitonin (CT Receptor). There are two common isoforms, CT Receptor *a*, insert-negative, and CT Receptor *b* insert-positive [157]. In the context of the expression of the CT Receptor by malignant glioma cells, targeting the CT Receptor might provide an opportunity to overcome resistance to chemotherapeutics. Furthermore, an antibody (immunotoxin) against the CT Receptor was created. The elevated membrane CT Receptor expression in high-grade glioma cell line U78MG increased its sensitivity to the immunotoxin [158]. On the other hand, the responses to CT receptor ligands (human CT, salmon CT, rat amylin) were reported in four high-grade glioma cell lines (WK1, JK2, SB2b, PB1) [159]. The CT Receptor was found non-functional in three of them. As possible reasons for inactivation were AS splicing, a switch to the CT Receptor *b* isoform or other mutations [159]. It was concluded that targeting the CT Receptor by immunotoxin is expected to provide an additional “knife”, in combination with traditional therapy of GBM [160]. The Glycogen Synthase Kinase-3 gene (GSK3) is responsible for a regulatory Ser/Thr protein kinase that exists in two distinct isoforms, GSK3α and GSK3β [161]. Both isoforms are inversely regulating hnRNPA1 (RNA binding protein) expression, subsequently affecting AS in glioma. HnRNP A2 and hnRNP B1 are proto-oncogenes that induce in vitro malignant transformation in glioma, most likely via aberrant splicing. It was found that silencing GSK3α activates hnRNPA1 and increases anti-apoptotic splice variants. It was suggested that inhibiting GSK3β along with the GSK3α recovery might be the best strategy to suppress glioma progression [155]. While AS was studied extensively in the context of molecular classification, prognosis or as a rich source of targets for therapy, few studies analyzed splice variants of genes related to chemotherapy failure or to sensitivity to drug treatment in glioma. The epidermal growth factor receptor (EGFR) gene encodes a primary mRNA transcript, referred to as EGFR variant 1 (EGFRv1) and three other alternatively spliced mRNAs that encode truncated forms of the receptor (isoforms *b*, *c*, and *d*). They lack the cytoplasmic tyrosine kinase domain [162]. The existence of truncated EGFR isoforms in glioma and its significance in therapeutic management are not clarified yet. Some authors assume that non-functional receptors result in the failure of therapeutics which target the EGFR extracellular domain [163], others report that the truncated EGFR isoforms may be predictive of the therapeutic response to Gefitinib in adenocarcinomas [164]. It was determined that glioma express EGFR transcripts other than EGFRv1 mRNA, that encode the full-length and functional EGFR isoform *a*. It was suggested that the EGFR isoforms might be a potential target for diagnostic and therapeutic strategies [165]. After activation of EGFR, Phosphatidylinositol 3-kinase (PI3K)/Akt/rapamycin-sensitive (PI3K/Akt/mTOR) signal pathway plays role in the genesis and development of glioma [166]. Inhibitors targeting EGFR and PI3K/ Akt/mTOR pathways are investigated in preclinical and clinical studies [167]. A resistance and decreased efficacy of the treatment was observed [168,169]. It was concluded that studying splicing events in this pathway will provide an alternative approach against resistance to therapy. It was demonstrated that drugs which target the general splicing process efficiently destroy cancer cells.

Recently, efforts are focused on the manipulation of MKNK2 (MAPK Interacting Serine/Threonine Kinase 2 gene) AS with the aim to inhibit cancer cell growth and survival [170]. The AS of pre-mRNA results in two proteins with different C-termini—longer Mnk2a and shorter Mnk2b having antagonistic effects. It was shown that down-regulated Mnk2a served as a tumor suppressor by inducing the transcriptional activation of its target genes and p38–MAPK-mediated cell death [171]. In accordance, the modulation of MKNK2 AS by a set of Splice–Switching antisense oligonucleotides (SSOs), increased Mnk2a levels which led to inhibition of cancer cell lines growth. Glioblastoma cells sensitized to chemotherapy and inhibited glioblastoma tumor growth in vivo was observed as a result [170]. Moreover, suppression of Protein arginine methyltransferase 5 (PRMT5) by two orthogonal-acting inhibitors (GSK591 or LLY-283), was shown to reduce the growth of GBM stem cell cultures derived from 46 patients [172]. The authors demonstrated that the PRMT5 inhibition was followed by massive perturbation of splicing across the transcriptome, mostly having an effect on cell-cycle related genes. Using the opportunity of RNA-seq data of TCGA, a comprehensive analysis of splicing events associated with prognosis in patients with glioma was performed, as well as new ideas for its diagnosis and treatment were proposed [173]. Furthermore, a new classification of low-grade glioma based on AS events was suggested [173]. It was indicated that differential expression of alternative exon usage (type of splicing events) could be applied as a biomarker for GBM [174]. An increasing number of studies investigated AS as an additional tool for glioma classification. The establishment of a tumor database facilitates bioinformatics analysis on splicing events and the outcome of cancer patients [175]. In support of the above the first methodologically profiled prognostic models were proposed. They have a satisfactory prediction value based on 69 and 88 prognostic events in low-grade glioma and GBM cohorts, respectively [176].

## 7. Metastatic Colorectal Carcinoma

Metastatic colorectal carcinoma (mCRC) is one of the leading causes of cancer related deaths worldwide [177]. About 25% of the patients with CRC have liver metastases at the time of diagnosis, and another 50% will develop such during the follow-up [178,179]. The initial or acquired drug resistance is the leading cause for the development of mCRC [180]. In addition, the conventional monitoring such as cross-sectional imaging and measurement of serum CEA and CA 19-9 appear to be insufficient as these biomarkers hold several drawbacks related to the reports of both false positive and false negative results [181,182]. To develop better strategies for treatment of mCRC and improve patient’s outcome, novel, more informative, less invasive prognostic biomarkers are needed. The establishment of non-invasive diagnostic tools, such as AS alterations, provides the opportunity to predict the individual response to chemo- or targeted therapy. Angiogenesis is one of the major hallmarks of cancer development and progression. As a result of AS of vascular endothelial growth factor A (VEGFA), several VEGFAxxxb antiangiogenic isoforms are produced. It was found that the antiangiogenic isoform VEGFA145b may predict resistance to Bevacizumab. Interestingly, the obtained results regarding Bevacizumab-required resistance are specific for right-sided primary tumors and not for the left-sided ones [183]. It was shown that VEGF isoforms determined CRC susceptibility to antiangiogenic therapy [184]. In addition, the intracellular protein T-cell Intracellular Antigen (TIA-1) alters the processing of VEGF-A mRNA [185]. Zadeh et al. found that in WT and Mut K-Ras CRC tissues and cells, a truncated protein TIA-1 (sTIA-1) form was expressed. Moreover, the depletion of sTIA-1 or the induction of full-length TIA-1 (flTIA-1) led to the expression of the anti-angiogenic VEGF isoform VEGF-A165b. Further, flTIA-1 has the ability to bind to VEGF-A165 mRNA and increase its translation, whereas sTIA-1 prevents this interaction. The in vivo experiments in nude mice showed that xenografted colon cancer cells over-expressing flTIA-1 formed smaller, less vascular tumors than those expressing sTIA-1. However, flTIA-1 expression inhibited the effect of anti-VEGF antibodies. The authors conclude that AS of TIA-1 can regulate isoform specific expression of VEGF, thus underlining the complexity of the angiogenic profile in CRC and the followed resistance to anti-angiogenic therapy [184].

Spleen tyrosine kinase (SYK) acts as a potential tumor suppressor in CRC and can exist in two isoforms: full length SYK [SYK(L)] and short form SYK [SYK(S)] [186]. It was observed that the lentiviral-induced SYK(L) overexpression had a suppressive effect on the proliferation and metastasis of HCT 116 cells, whereas the overexpression of SYK(S) did not. Moreover, both SYK(L) and SYK(S) increased the sensitivity of the cells to 5-fluorouracil (5-FU), suggesting that SYK(L) and 5-FU produces a significant synergistic effect on CRC cell proliferation, while SYK(S) has an effect on modulating 5-FU sensitivity of CRC. The affected genes, the mutation type and its relation to drug resistance in mCRC are presented on Table 5.

## 8. Hematologic Malignancies

Hematologic malignancies account for 6–10% of all cancers and encompass a large group of blood neoplasms including different types of lymphoma, multiple myeloma, as well as myeloid and lymphoid leukemia [50,187]. The prognosis in this heterogeneous group of diseases ranges from nearly 90% overall survival rates in acute promyelocytic leukemia [188] to less than 6% after early relapse of acute lymphoblastic leukemia [189]. While there are certain recurrent genetic mutations in leukemia, hematologic malignancies remain largely heterogenic. There is a myriad of molecular subtypes with more than 20 suggested subgroups only in pediatric B-cell leukemia [190]. The lack of overlapping mutations may be in part due to underestimation of the role of AS. In support of this notion, EZH2 aberrations in acute myeloid leukemia (AML) may be in fact three times more frequent if we consider inactivation due to AS [191]. The role of AS of oncogenes and tumor suppressors as well as particular splice factor mutations for leukemogenesis are widely-recognized [192,193]. Even though most of these mutations are not studied functionally and remain of unknown impact, many genetic aberrations in splicing factors correlate with poor survival rates. For instance, lesions in Splicing factor 3B1 (SF3B1) occur in 5–17% of chronic lymphocytic leukemia (CLL) [194] and in up to 46% of chronic myelomonocytic leukemia (CMML) patients [195], respectively, and are implicated in worse prognosis. There is mounting evidence that aberrant AS is key to drug response too [193,196].

### 8.1. AS in Lymphoid Malignancies

Methotrexate (MTX), a folic acid analogue, is a key component in the standard chemotherapy for acute lymphoblastic leukemia (ALL). Aberrations in the folylpoly-γ-glutamate synthetase (FPGS), an enzyme which converts MTX in the cell to a more potent inhibitor of folate metabolism and DNA synthesis (MTX polyglutamate), are well-established as a common resistance mechanism in ALL [197]. Several studies indicate that changes in AS of the FPGS gene (exon skipping or intron retention) constitute MTX resistance mechanisms in T-ALL where exon 12 is skipped [198], and in childhood ALL (both B- and T-cell) where intron 8 is partially retained [199]. Importantly, these splice variants can result in two major types of incapacitating events—truncations of FPGS (following skipping of exons 5, 10, and 12, and retentions of intron 6 and 8), and alterations in the 3D conformation of the enzyme (as a consequence of exon 2 and 6 skipping). Either mechanism of FPGS inactivation is ultimately due to loss of glutamate binding and thereby inability to produce the more stable and potent MTX polyglutamate in the cell. Such splice variants and the concomitant aberrations in FPGS are directly implicated as a rescue mechanism to anti-folate treatment in ALL [200]. FPGS splicing mutations, most likely being indicative of other unknown AS lesions, were correlated to resistance to other chemotherapeutics like Dexamethasone, Mitoxanthrone, and Prednisolone as well [199]. More direct evidence of AS in resistance to glucocorticoids (GCs) comes from a particular isoform of the GC receptor itself. The GRβ splicing variant (with partial splicing of exon 9) fails to bind GC, cannot regulate the transcription of genes with GC-response elements, and it can act as a dominant negative isoform when dimerized with the canonical and active glucocorticoid receptor (GR) GRα isoform [38]. GRβ expression was implicated in poor GC-response in ALL [38,201,202,203,204]. Another isoform, GR-P, missing exons 8 and 9, is less well studied, but its expression may also be implicated in GC resistance [205]. Of note GR AS variants (with deletions of exons) were isolated from a GC-resistant multiple myeloma patient sample [206] suggesting common mechanisms of drug resistance across multiple malignancies. Comparing GC-sensitive (*n* = 15) to GC-resistant (*n* = 23) primary pediatric ALL samples, Sciarrillo et al. described hundreds of differentially spliced events, which may be related to drug response. Some of the more interesting genes with AS included *HSP90AA1*, which is responsible for the correct maturation and nuclear transport of GRs, and *SGK1*, which is a direct GR target and has antiapoptotic functions. What the authors also found was AS in splice factors such as U2AF1, and several HNRNPs and SRSFs, which can alter global AS in the cell and thereby drug response and cancer progression. Importantly, this study also points to a more general set of splicing alterations that could make cells resistant to any chemotherapeutic agent—AS of genes involved in apoptosis (TP53β and BAX), in survival and proliferation pathways like MAPK, NFk-B and JNK, in oxidative phosphorylation (and production of ROS which may trigger apoptosis), and others [39]. Mutations in a splicing factor, which are likely to result in hundreds or even thousands of alternatively spliced isoforms, correlate with poor drug response in lymphoid malignancies. In 10 out of 59 CLL patients (17% of the cohort) refractory to the purine analogue Fludarabine, SF3B1 mutations were found by targeted sequencing. In samples taken at diagnosis, the authors described such aberrations in only 17 out of 301 patients (5% of the cohort) with 5 out of 17 patients showing primary lack of response to Fludarabine. Importantly, the authors also demonstrated that lesions in this gene correlate with worse overall survival and are an independent prognostic marker [194]. Of note, SF3B1 mutations, even if at lower rate, were described in myelodysplastic syndrome (79/582 or ~7%) [207] and Richter syndrome with diffuse large B-cell lymphoma (6%) [194], pointing to a recurrent role in the development and/or the progression of hematologic malignancies. The mechanisms of action behind the observed drug resistance following SF3B1 mutations are largely unknown. However, te Raa and colleagues found that CLL cells harboring such a genetic lesion exhibit defective DNA damage response and apoptosis independent of p53 [208]. Lastly, AS can also impair immunotherapy. Sotillo et al. detected that mutations in the splicing factors SRSF3 can result in altered exon 2 splicing of CD19 rendering CART-19 cell therapy ineffective in B-cell ALL as well [209].

### 8.2. AS in Myeloid Malignancies

A recent meta analysis in acute myeloid leukemia (AML) using TCGA transcriptomics data found that nearly 2000 genes undergo AS and these events correlate strongly to overall survival [210]. Similarly, multi-cohort analysis (of more than 700 patients) of RNA-seq identified a specific AS prognostic signature for risk stratification in AML [211]. Another large study using RNA-seq of 1258 myeloid neoplasm patients and 63 control samples revealed common mutations (in ~60% of patients) in 7 splicing factors. They include SF3B1, SRSF2, and U2AF1, and lead to more than 17,000 different splicing events affecting cellular processes like proliferation [212]. Besides overall survival, alternative exon usage (AEU) of more than 3000 genes was shown to distinguish between chemo-sensitive and refractory primary AML samples. The role of AS in drug resistance in myeloid malignancies was confirmed by initially discovered in 24 patients and then 70% of the AEU was validated in 152 patients [213]. The link between response to pyrimidine analogues and AS was very well-documented in AML. Veuger and colleagues demonstrated that cytarabine-resistant AML patients (7 out of 12) harvest multiple splice variants of the enzyme which activates cytarabine—deoxycytidine kinase (dCK). Such isoforms are absent in normal bone marrow CD34+ cells or in samples from good-responders. Importantly, functional validation of dCK missing exons 2–6 (in different combinations) showed that these AS forms may not even be translated to protein. This leads to inability to convert Cytarabine to its active form in the absence of a wild type transcript to create a functional enzyme [214,215]. Besides dCK exon skipping, another mechanism for Cytarabine resistance in vitro is indicated by splice variants in the ENT1 nucleoside transporter which hinder sufficient uptake of pyrimidine analogues [216]. A study in AML cell lines and 152 patient samples revealed distinct patterns of exon expression between drug-sensitive and resistant cells. Namely, Mohamed et al. found that skipping of Exon 2 in TET2 correlates to Cytarabine resistance [213]. Resistance to another pyrimidine analogue chemotherapeutic, Sulfasalazine, was shown to arise in vitro (in two AML cell lines) from unspliced thymidine phosphatase (TP), which is the key enzyme that ultimately converts this chemotherapeutic agent to its active form—5-fluoruracil [217]. As mentioned for lymphoid malignancies, splice variants of anti-apoptotic proteins may be indirectly involved in drug resistance as well. Expression of different Bcl-2 isoforms was described in quiescent CML cells and linked to TKI-resistance [218]. The ratio of two isoforms of the anti-apoptotic protein Survivin (Survivin-2B/ΔEx2) was correlated to refractory disease in childhood AML (*n* = 306 patients) [219]. Similarly, the expression of neuronal apoptosis inhibitory protein (NAIP) relative to its isoform NAIP-deltaEx10–11 in multi-drug resistant HL-60 cells is increased and provides a likely chemotherapy escape mechanism [196,220]. On the other hand, AS may sometimes lead to isoforms that correlate to better prognosis. This is exactly the case with two variants of p53 in AML—p53β and γ—which are markers for better chemotherapy response (to Doxorubicin in particular) [221]. Such truncated isoforms of p53 have been linked to altered promoter binding and enhanced activity of the p21 tumor suppressor and of pro-apoptotic genes such as BAX [222]. Similarly, loss of a particular isoform of the pro-apoptotic protein Bcl-x (Bcl-xs), is associated with relapse and worse survival [223]. Table 6 presents data on drug resistance and AS in hematologic malignancies.

**Figure 3:**
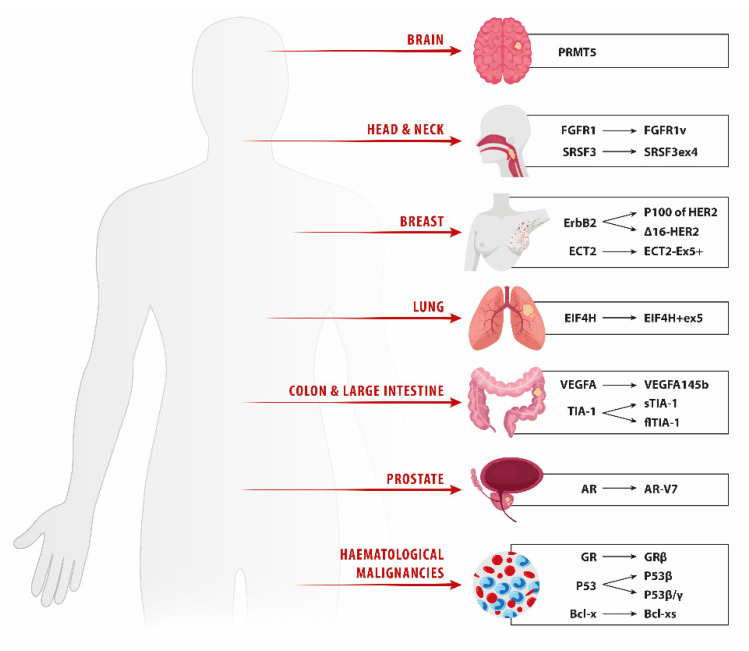
Cancer-related genes undergoing AS and their significance for drug resistance. The figure summarizes representative AS events that contribute to chemotherapy failure in different cancers. PRMT5 (Protein Arginine Methyltransferase 5); FGFR1 (Fibroblast Growth Factor Receptor 1); SRSF3 (Serine and Arginine Rich Splicing Factor 3); ErbB2 (Erb-B2 Receptor Tyrosine Kinase 2); EIF4H (Eukaryotic Translation Initiation Factor 4H); VEGFA (Vascular Endothelial Growth Factor A); TIA-1 (TIA1 Cytotoxic Granule Associated RNA Binding Protein); AR (Androgen Receptor); GR (Glucocorticoid receptor); P53 (Tumor Protein P53); Bcl-x (BCL2 Apoptosis Regulator).

A representative set of AS events that contribute to tumor development, progression, and drug resistance in different cancers is shown on Figure 3.

## 9. Conclusions and Future Perspectives

The connections between AS and cancer are proven by the existence of alternatively spliced isoforms which drive the oncogenic process or serve as therapeutic targets. In evolutionary aspect, we can hypothesize that cancer cells utilize alternative RNA splicing to survive, proliferate, and gain therapeutic resistance. A number of isoforms could be identified and further used as biomarkers of disease progression or as predictors of response to therapy. Although splicing aberrations confer new peptide variants that could potentially serve as neo-antigens for the immune system to cope with tumor formation, it should be noted that the process of mutagenesis is comprised of multi-dimensional phenomena. They exert effects on coding and non-coding levels, including epigenetic control, gene transcription and regulation, ultimately resulting in prolonged cell survival in low profile immune cancer phenotype. Different ncRNAs such as miRNAs (microRNAs), lncRNAs (long noncoding RNAs), circRNAs (circular RNAs), and snRNAs (small nuclear RNAs) play a pivotal regulatory role that mediate cancer progression through AS [224]. Their mechanisms modulate a plethora of molecular targets to regulate cis-acting elements, trans-acting factors, or pre-mRNA transcription at multiple levels, leading to tremendous alterations in the AS process and generating alternatively spliced isoforms in cancer cells.

The mechanisms of therapy resistance are still one of the biggest obstructions in cancer treatment. Recently, a few circular RNAs circRNAs such as CiRS-7, circRNA-MTO1, hsa_circ_0023404, CircRNA_101505 were reported to play crucial roles in the chemotherapy resistance [225]. These single-stranded RNA molecules resulted by alternative mRNA splicing are promising for overcoming the resistance in cancer [226]. However, studies of circRNAs in cancer resistance to radiation and chemotherapy, are still at the preliminary level. Therefore, to cope with such a complexity, a more sophisticated approach should be pursued. A combination with gene silencing and application of small molecules together with immune checkpoint inhibitors is most likely to be used, in order to address the complex nature of cancer plasticity.

The AS events described in the present manuscript are prime targets for future studies that might lead to the development of different splicing modulators. Thus, the application of systematic and genome-wide mapping approaches such as next-generation sequencing (NGS) in cancers will lead to the identification of new splicing events or splicing factors that are related to therapy resistance in cancer. In this regard, the achievements in cancer genomics will pave a new road in the aspect of establishment of novel diagnostic and therapeutic strategies towards cancer precision medicine.

## Figures and Tables

**Table 1:** AS and effects on drug resistance in lung cancer.

Malignancy	Gene	Splice Variant	Mutation Type	Drug Resistance	Biological Function	Reference
NSCLC	PTPMT1	Exon skipping	SRSF1 target	Radioresistance	Promotes phosphorylation of AMPK PTEN-like mitochondrial phosphatase	[47]
	BIM	Alternative splicing	SRSF1	Imatinib		[48]
	U2AF1		Loss of function	Gemcitabine, glucocorticoids	Target of the splicing factor quaking (QKI)	[39]
	SRSF1 metastasis-associated lung adenocarcinoma transcript 1 (MALAT1)	SRSF1 + ex2 (MCL1L) Alternative 5′SS of exon 2 (BCL-XL)	N/A	Gemcitabine	MNK2 splicing; MCL1 member of BCL2 family splicing to MCL1L long anti-apoptotic variant	[41,42]
LUAD	EIF4H	EIF4H + ex5	Gain of function	Resistance to PI(3)K/AKT/mTOR inhibitors (e.g., AZD8055, BEZ235)	Cancer related genes translation	[49]

**Table 2:** AS and effects on drug resistance in breast cancer.

Malignancy	Gene	Splice Variant	Drug Resistance	Biological Action	Reference
Breast Cancer	HER2	P100 of HER2 Δ16-HER2 d16HER2	Trastuzumab	SRSF3 and hnRNPH1 are associated with splicing regulation of Δ16-HER2. d16HER2 influences tumor initiation and aggressiveness, cancer stem cell properties, epithelial-mesenchymal transition and HER2-positive breast cancer cell susceptibility to trastuzumab	[55,57]
	ECT2	ECT2-Ex5+	Doxorubicin	ZRANB2 and SYF2-mediated AS programs converging on ECT2 act in drug resistance	[60]

**Table 3:** AS and effects on drug resistance in prostate cancer.

Malignancy	Gene	Splice Variant	Mutation Type	Drug Resistance	Biological Function	Reference
Prostate Cancer	AR	AR-V7	Cryptique exon 3 inclusion, exon skip	ADT resistance—Enzalutamide, Abiraterone	Activate target gene expression	[84,85]
	SRSF3 SF3B2 U2AF2	Alternative splicing	Poison exon insertion	AR-V7 induction	Myc interaction and NEPC induction	[90,102,104]
	EZH2	EZH2 + ex14	Exon 14 inclusion (promoted by SF3B3)	AR induction	Enzalutamide	[115,119]

**Table 4:** AS and effects on drug resistance in head and neck squamous cell carcinoma.

Malignancy	Gene	Splice Variant	Mutation Type	Drug Resistance	Biological Function	Reference
Adenoid cystic carcinoma (HNSCC)	FGFR1	FGFR1v	Premature termination codon at position 147 of intronic segment	Dovitinib	Mediate FGF/FGFR1-independent function through the AXL/AKT signaling axis	[149]
OSCC	SRSF3	SRSF3ex4	Long isoform with exon 4 encodes a truncated SRSF3 protein	Paclitaxel	Increases the expression of c-Jun, cyclin D1, cyclin D3, CDC25A and E2F1, and accelerates cell growth	[58]

**Table 5:** AS and effects on drug resistance in mCRC.

Malignancy	Gene	Splice Variant	Mutation Type	Drug Resistance	Biological Function	Reference
mCRC	VEGFA	VEGFA145b	Differential splicing of the 3′ distal site of exon 8	Bevacizumab	Act as a reservoir of angiogenic growth factors in the tumor stroma	[183]
	TIA-1	sTIA-1 flTIA-1	Exon 5 exclusion leading to truncated protein	Anti-VEGF antibodies	Alters both co-transcriptional and post-transcriptional RNA processing	[184]

**Table 6:** AS and effects on drug resistance in hematologic malignancies.

Malignancy	Gene	Splice Variant	Mutation Type	Drug Resistance	Biological Function	Reference
CLL	SF3B1	SF3B1-ΔHEAT	Deletions in HEAT domains	Fludarabine	Splicosome factor	[194]
T-ALL, cALL (both T- and B-cell)	FPGS	FPGS-ES(12);IR(8)	Exon 12 skipping; intron 8 partial retention	Methotrexate Dexamethasone, Mitoxanthrone, Prednisolone	Intracellular modification of MTX	[198,199,200]
ALL	GR	GRβ	Downstream acceptor site in exon 9	Glucocorticoids	Inactive GC receptor; dominant negative isoform	[38]
	P53	P53β	Exon 9β inclusion	Glucocorticoids	Higher expression in resistant cells	[39]
AML	dCT	dCT-ΔEx2–6	Missing exons 2–6 (deletions)	Cytarabine	Enzyme, which activates Cytarabine	[214,215]
	TET2	TET2-ES(2)	Skipping of exon 2	Cytarabine		[213]
	P53	P53β/γ	Alternative splicing of exon 9β or 9γ	Doxorubicin	Better prognosis/active tumor suppressor	[221]
	Bcl-x	Bcl-xs	Alternative splicing	Multiple drugs	Altered apoptosis; loss of bcl-xs leads to worse RFS and OS	[223]

ES-exon splicing; IR-intron retention.

## Data Availability

Not applicable.
